# The interplay between nonalcoholic fatty liver disease and atherosclerotic cardiovascular disease

**DOI:** 10.3389/fcvm.2023.1116861

**Published:** 2023-05-02

**Authors:** Alexandra C. Finney, Sandeep Das, Dhananjay Kumar, M. Peyton McKinney, Bishuang Cai, Arif Yurdagul, Oren Rom

**Affiliations:** ^1^ Department of Pathology and Translational Pathobiology, Louisiana State University Health Shreveport, Shreveport, LA, United States; ^2^ Department of Molecular and Cellular Physiology, Louisiana State University Health Shreveport, Shreveport, LA, United States; ^3^ Division of Liver Diseases, Department of Medicine, Icahn School of Medicine at Mount Sinai, NY, United States

**Keywords:** atherosclerosis, animal models, biomarkers, nonalcoholic fatty liver disease, nonalcoholic steatohepatitis, pathophysiology, therapeutics

## Abstract

Therapeutic approaches that lower circulating low-density lipoprotein (LDL)-cholesterol significantly reduced the burden of cardiovascular disease over the last decades. However, the persistent rise in the obesity epidemic is beginning to reverse this decline. Alongside obesity, the incidence of nonalcoholic fatty liver disease (NAFLD) has substantially increased in the last three decades. Currently, approximately one third of world population is affected by NAFLD. Notably, the presence of NAFLD and particularly its more severe form, nonalcoholic steatohepatitis (NASH), serves as an independent risk factor for atherosclerotic cardiovascular disease (ASCVD), thus, raising interest in the relationship between these two diseases. Importantly, ASCVD is the major cause of death in patients with NASH independent of traditional risk factors. Nevertheless, the pathophysiology linking NAFLD/NASH with ASCVD remains poorly understood. While dyslipidemia is a common risk factor underlying both diseases, therapies that lower circulating LDL-cholesterol are largely ineffective against NASH. While there are no approved pharmacological therapies for NASH, some of the most advanced drug candidates exacerbate atherogenic dyslipidemia, raising concerns regarding their adverse cardiovascular consequences. In this review, we address current gaps in our understanding of the mechanisms linking NAFLD/NASH and ASCVD, explore strategies to simultaneously model these diseases, evaluate emerging biomarkers that may be useful to diagnose the presence of both diseases, and discuss investigational approaches and ongoing clinical trials that potentially target both diseases.

## 1. Introduction

Despite the remarkable advances in interventional therapeutics, decades of basic science and clinical research, atherosclerotic cardiovascular disease (ASCVD) remains the leading cause of death worldwide (1). While the overarching pathoetiology largely arises from dyslipidemia, the imbalance of cholesterol and triglyceride lipids, numerous comorbidities complicate and exacerbate ASCVD (1). Of particular significance are metabolic- and obesity-related diseases, which have globally increased prevalence since the 1970s (2). Nonalcoholic fatty liver disease (NAFLD) and nonalcoholic steatohepatitis (NASH) are also strongly associated with the metabolic syndrome (3), which currently afflicts approximately 90% of obese patients (4) and approximately 55% of individuals with type 2 diabetes (T2D) (5). Globally, the incidence of NAFLD has increased from 25% in 2005 to 32% today (6), highlighting an alarming trend in rising NAFLD burden. Despite this, no FDA-approved drug exists in the treatment of NAFLD/NASH. While NAFLD is associated with increased risk of liver-related mortality, the most common cause of death in patients with NAFLD, particularly those with the more severe NASH, is cardiovascular disease (CVD) (7–12). This, combined with the rising prevalence of both ASCVD and NAFLD has led to extensive discussion of the relationship between these two diseases. In 2022 alone, the increasingly transparent relationship between NAFLD/NASH and ASCVD has piqued interest between multiple scientific fields of expertise (13–17), culminating in a scientific statement from the American Heart Association (8).

**Figure 1:**
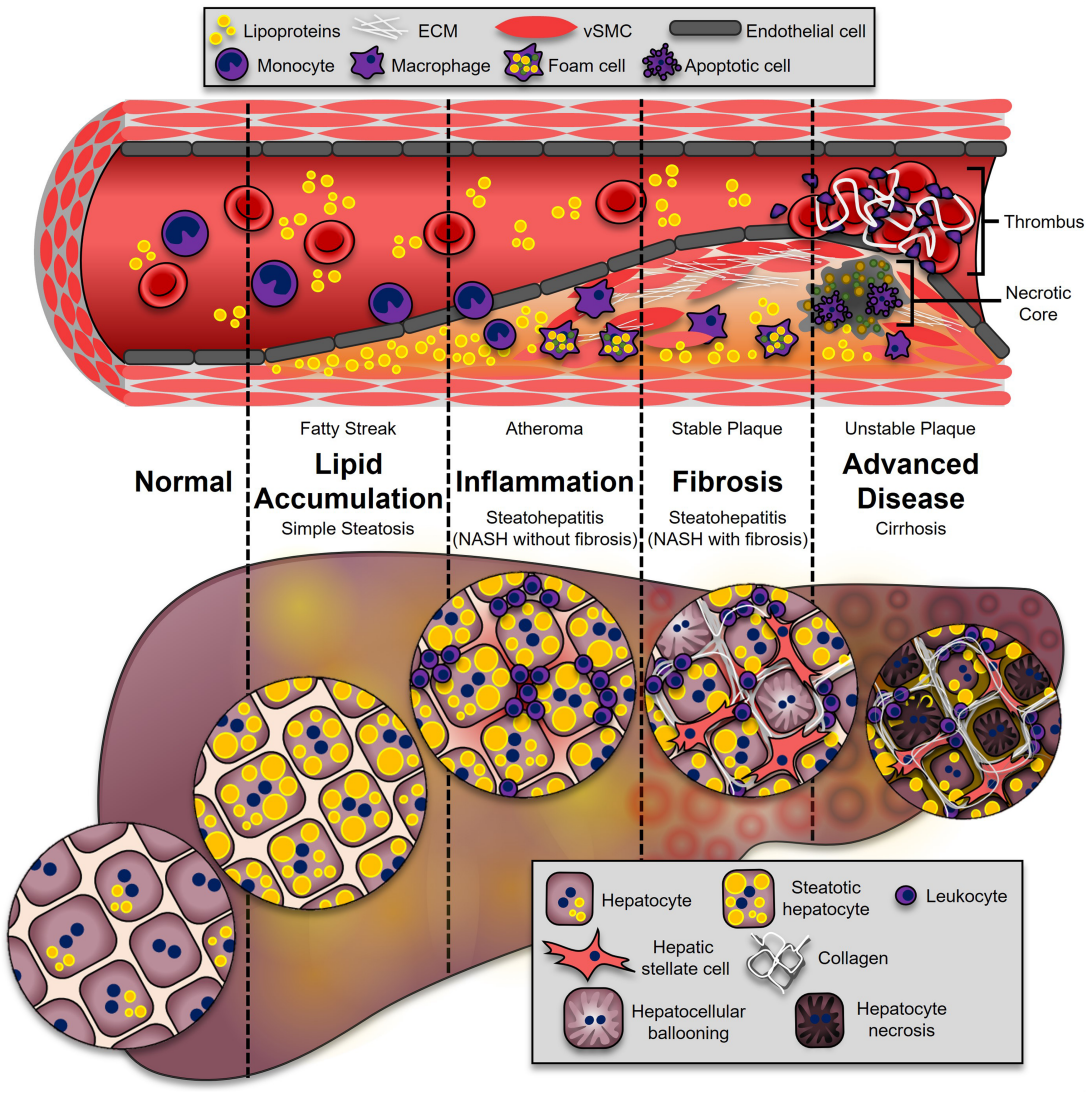
Progression of ASCVD and NASH. The onset of both ASCVD and NASH begins with dysregulated lipid metabolism, leading to their accumulation in the neointimal region of the artery (fatty streak), or the hepatocytes (simple hepatic steatosis). This process enhances inflammatory pathways in both diseases. During atherosclerosis, leukocytes adhere and transmigrate into the developing plaque, where they secrete additional cytokines and chemokines (atheroma). In the liver, leukocytes from the circulation accumulate, leading to NASH (NASH without fibrosis). These immune cells secrete soluble factors to activate collagen-producing cells: synthetic vascular smooth muscle cells (vSMCs) in atherosclerosis (stable plaque), and hepatic stellate cells in the liver (NASH with fibrosis). The most advanced stages of disease are associated with higher mortality. In atherosclerosis, advanced plaques with a large necrotic core and thin fibrous caps are prone to rupture (unstable plaque), which is highly thrombogenic. In the liver, excessive fibrosis and cell death leads to irreversible damage and loss of liver function (cirrhosis).

Despite this acknowledgement, the specific mechanisms regulating the onset, crosstalk, and exacerbation of NAFLD and ASCVD remain unclear. The reasons for this are multifactorial: (1) there is no single established model to study NAFLD/NASH and ASCVD simultaneously, (2) since most patients with NAFLD/NASH and ASCVD are asymptomatic, diagnosis is often incidental and limited to routine blood screening (e.g., plasma lipids, liver transaminases) (18), calcium imaging (19), or less routinely, biopsy (20), and (3) clinical trials have remained limited in targeting either NASH or atherosclerosis, thus, it is unknown whether current clinical trials for NASH affect cardiovascular outcome or vice versa. For example, obeticholic acid, the most advanced drug candidate for NASH, causes hyperlipidemia, raising concerns about the possible adverse consequences on ASCVD (21). Furthermore, the effect of traditional therapies for ASCVD, e.g., statins, on NASH has shown inconsistent results in improving histological features of NASH (22, 23). Thus, strategies that simultaneously interrogate therapies for both NASH and ASCVD are necessary. This review will provide insight into each of these limitations, offering a comprehensive and current summary of our understanding regarding the relationship between NAFLD/NASH and ASCVD (Figure 1). Below, we (1) summarize the molecular drivers that regulate ASCVD and NAFLD/NASH, (2) discuss which animal models should be considered for evaluating translational interpretation of preclinical findings, (3) review emerging biomarkers for both NASH and atherosclerosis that may also serve as therapeutic strategies, and (4) examine potential limitations and caveats for the concurrent treatment of both NASH and ASCVD.

## 2. Pathophysiology of ASCVD and NAFLD/NASH

### 2.1. Mechanisms driving the initiation and progression of atherosclerosis

Most cases of myocardial infarction and stroke are caused by atherosclerosis, the fibrofatty plaques in the arterial branch of the vascular tree (24). The formation of atherosclerotic plaques is driven primarily by the deposition of apolipoprotein (Apo)-B-containing lipoproteins in the subendothelial spaces of the intima that subsequently drive maladaptive, non-resolving inflammation (25). Thus, individuals with familial hypercholesterolemia, particularly in the low-density lipoprotein (LDL) fraction, are disposed to developing atherosclerotic plaques at an early age (26). Other risk factors include insulin resistance and metabolic syndrome (27). Advanced atherosclerotic plaques contain vast amounts of extracellular matrix (ECM) proteins, calcium minerals, and a large necrotic core formed from the death of lipoprotein-rich monocyte-derived macrophages. These advanced atherosclerotic plaques can impede blood flow to downstream tissues through occlusion of the vessel lumen, causing symptomatic ischemia (24). More frequently however, atherosclerotic plaques rupture and leak the highly thrombogenic contents from the necrotic core into the lumen, resulting in an occlusive thrombus. Deaths from ASCVD were declining over the last two decades as treating more individuals for high LDL (∼28% in 1999–2002 to ∼48% in 2005–2008) resulted in twice as many individuals successfully lowering their circulating LDL-cholesterol from ∼15% to ∼33% (28). Despite the advent of potent cholesterol-lowering medicines, such as statins and anti-proprotein convertase subtilisin/kexin type 9 (PCSK9)-blocking antibodies, ASCVD remains the leading cause of death worldwide. More troubling is the recent trend that life expectancy growth has begun to decline, with a substantial rise in CVD deaths having the most impact (29). Thus, a deeper understanding of the cellular and molecular mechanisms driving atherosclerosis is necessary to conceive novel therapeutic strategies.

#### 2.1.1. Endothelial cell activation

LDL particles accumulate in the subendothelial intima due to increased endothelial cell permeability caused by disturbed blood flow (30). Apart from the antioxidant environment normally provided by the blood, LDL particles become oxidized (ox-LDL) by unmitigated reactive oxygen species (ROS) production, leading to its uptake by scavenger receptors (31, 32). Unlike the LDL receptor (LDLR), scavenger receptors undergo positive feedback that maintains persistent cellular uptake of ox-LDL (33). Endothelial cells that take up ox-LDL activate the proinflammatory transcription factor nuclear factor-*κ*B (NF-*κ*B) that drive the expression of adhesion molecules, such as intercellular adhesion molecule 1 (ICAM1) and vascular cell adhesion molecule 1 (VCAM1) (34). These adhesion molecules presented on the apical surface of endothelial cells bind circulating leukocytes and promote their entry into the vessel wall. The role of endothelial cell activation in promoting atherosclerosis is crucial, as atherosclerosis formation tends to only occur at sites of disturbed blood flow, such as curvatures, branch points, and bifurcations, and experimental strategies that prevent endothelial cell activation prevent atherosclerosis formation (30).

#### 2.1.2. Vascular smooth muscle cell dedifferentiation and altered macrophage functions

Vascular smooth muscle cells (vSMCs) regulate blood pressure and vessel integrity under normal conditions (35). However, during early atherosclerosis, vSMCs undergo dedifferentiation whereby they lose canonical vSMC markers, such as α smooth muscle actin (αSMA) and transgelin (SM22), and reignite signaling pathways associated with development (36). Furthermore, dedifferentiated vSMCs begin to migrate, proliferate, and synthesize ECM proteins, thereby expanding the growing lesion towards the lumen of the vessel. Interestingly, vSMCs produce a panoply of ECM proteins that can retain growth factors, cytokines, and chemokines (35). Whereas soluble growth factors and cytokines transmit potent signals rapidly, matrix-bound and immobilized factors resist internalization and degradation, sustaining their signaling capabilities and promoting fibroproliferative remodeling (37).

After endothelial cells are activated in regions of disturbed flow, monocytes infiltrate the subendothelial intima, where they differentiate into macrophages. These monocyte-derived macrophages ingest rampant amounts of ox-LDL, transforming them into cholesterol-rich “foam cells” and compromising their beneficial immune cell functions (25). Macrophages are also highly susceptible to cell death owing to the intrinsic lipotoxic properties of ox-LDL that drive endoplasmic reticulum (ER) stress, resulting in their eventual death and release of damage-associated molecular patterns (DAMPs) in the surrounding microenvironment (38). Through various mechanisms, surrounding macrophages lose their ability to clear dying cells (termed “efferocytosis”), substantially expanding necrotic core areas and impairing the production of pro-resolving mediators, such as interleukin (IL)-10 and transforming growth factor beta (TGFβ) (39, 40). Importantly, experimental strategies to restore efferocytosis in settings where it fails, mitigate atherosclerosis and even promote its regression (41–43).

#### 2.1.3. Consequences of unmitigated atherosclerosis progression

Most acute cardiovascular events leading to myocardial infarction and stroke are caused by plaque rupture. During this process, highly thrombogenic material from the necrotic core, which is particularly rich in tissue factor, are released into the vessel lumen (24). Atheromas with relatively large necrotic cores and thin fibrous caps have often been considered “vulnerable” plaques, whereas “stable” plaques have much thicker fibrous caps (44). Macrophages and vSMCs are particularly sensitive to ox-LDL and undergo cell death, forming necrotic cores. An imbalance in fibrogenesis vs. fibrolysis impedes vSMC-dependent ECM synthesis and assembly and drives thinning of the protective fibrous cap. Inflammatory cells activated in the atherosclerotic milieu also possess robust levels of collagenases that degrade the collagen-rich fibrous cap. Structural weakening of the fibrous cap results in interfacial debonding, characterized as the physical separation of fibrillar matrix (45, 46). Notably, this phenomenon is frequently observed in ruptured atheromas (45, 46).

### 2.2. NAFLD: Onset, progression, and molecular drivers

NAFLD represents a range of liver pathologies beginning with excessive accumulation of lipids, particularly triglycerides, in hepatocytes (47). Additional findings of enhanced cytokine and chemokine production, inflammatory cell recruitment, and hepatocyte death characterize NASH, which may progress into fibrosis, cirrhosis, and liver failure. Importantly, NAFLD is emerging as a leading cause of liver disease, with 20%–30% of the individuals progressing to cirrhosis due to NASH (48, 49). Cardiometabolic disorders, such as insulin resistance and the metabolic syndrome, are risk factors contributing to the progression of NASH (50).

#### 2.2.1. Hepatic steatosis and lipotoxicity

Increased caloric consumption is one of the leading causes of NAFLD, as excessive substrate overload or dysfunction in the ability of adipose tissue to store fats results in lipolysis (51). Consequently, circulating free fatty acids increase and are then taken up by secondary storage organs, particularly the liver, through fatty acid transport protein 5 (FATP5) and the scavenger receptor CD36 (52, 53). This stimulates signaling pathways that ultimately drive intrahepatic triglyceride accumulation. In addition, *de novo* lipogenesis (DNL) promotes hepatic steatosis by converting carbohydrates into lipids. Thus, the three main pathways, (1) enhanced lipolysis from adipose tissue, (2) triglyceride synthesis from the dietary nutrients, and (3) the conversion of dietary sugars into fatty acids by DNL, drive hepatic steatosis. In this manner, the liver's capacity to adequately process carbohydrates and fatty acids become impaired, and the formation of toxic lipid species, such as lysophosphatidylcholines, diacylglycerols, and ceramides, takes place (51). Consequently, these lipotoxic lipid species elicit a robust unfolded protein response (UPR) and ER stress that promote inflammasome activation and cell death.

Excess accumulation of intrahepatic fatty acids drives ER stress, uncouples mitochondria, and elevates ROS production by the mitochondria (54). Consequently, Jun N-terminal kinase (JNK) becomes activated and promotes intrinsic apoptosis through a caspase-2-BID signaling pathway (55). Also, fatty acid conversion to triglycerides increases the expression of death receptors and their cognate ligands, tumor necrosis factor alpha (TNFα) and Fas, to stimulate extrinsic cell death. Intrinsic or extrinsic apoptosis leads to the release of DAMPs that crosstalk with either resident or recruited macrophages to stimulate toll-like receptor (TLR)-dependent expression of multiple proinflammatory cytokines and chemokines.

#### 2.2.2. Inflammation

A critical feature that distinguishes hepatic steatosis from NASH is the presence of hepatic inflammation, particularly of resident Kupffer cells and recruited monocyte-derived macrophages (56). Meta-analysis of RNA sequencing and single-cell RNA sequencing have revealed critical alterations in the myeloid compartment recruited to livers during NASH. Firstly, turnover and maintenance of embryonically-derived Kupffer cells are diminished during the progression of steatosis to NASH, likely due to lipotoxicity (57). Second, monocyte-derived macrophages recruited to the liver, which highly expresses *Trem2*, *Gpnmb*, *Cd9*, *Spp1,* and *Itgax*, genes associated with macrophages in NASH, accumulate in areas near desmin^high^ hepatic stellate cells, revealing their capability to crosstalk with hepatic stellate cells to drive hepatic fibrosis (58). Importantly, macrophages have been definitively proven to contribute to NASH, as depleting Kupffer cells from mice using liposomal clodronate, deleting the chemokine receptor C-C chemokine receptor type 2 (CCR2), or ablating bone marrow cells from mice using irradiation, mitigates the progression of steatosis to NASH (59–62).

Through a variety of mechanisms, macrophages in the liver exhibit a heightened state of inflammation and produce vast amounts of IL-1β (63). Consequently, peroxisome proliferator-activated receptor alpha (PPARα) becomes suppressed, and oxidation of fatty acids is impaired, ultimately leading to an accumulation of fatty acids (63). Fatty acids not only stimulate triglyceride production in hepatocytes, but they also stimulate inflammatory responses in liver immune cells (56). The saturated fatty acids, palmitate and laurate, drive IL-1β secretion by mediating NLRP3 inflammasome activation during NASH in a TLR2-dependent mechanism (56, 64, 65). Furthermore, palmitate activates NADPH oxidase 2 (NOX2) in hepatic macrophages and induces ROS production (66). Importantly, elevated levels of ROS directly stimulate TNFα expression. Also, macrophages from steatotic livers show enhanced production of toxic lipid mediators, particularly diacylglycerols and ceramides (56).

#### 2.2.3. Hepatic stellate cell activation and fibrosis

Persistent deposition of ECM proteins, such as collagens, in the liver drive cirrhosis and liver failure. Excluding CVD, liver fibrosis is the major cause of liver-related mortality in patients with NASH (47, 50). Therefore, hepatic fibrosis is among the most important endpoints in clinical trials. Hepatic fibrosis is largely mediated by the activation of non-parenchymal hepatic stellate cells that leads to their dedifferentiation towards a myofibroblast phenotype, enabling them to robustly synthesize and deposit ECM proteins (67). Evolutionarily conserved developmental programs, including Notch, hedgehog, and Hippo-YAP-TAZ, are “reawakened” in acute liver injury to stimulate hepatocyte regeneration (67). However, growing research in these pathways has revealed that they also critically drive hepatic fibrosis during NASH. For example, transgenic overexpression of Notch in hepatocytes leads to enhanced osteopontin secretion, enhancing fibrosis through hepatic stellate cell activation (68, 69). Consistently, hepatocyte-specific inactivation of Notch signaling protects mice from developing NASH-induced hepatic fibrosis (69). Whereas Hedgehog signaling is inactive in normal livers, it becomes reactivated during NASH and enhanced Hedgehog activity correlates with disease severity and fibrosis staging (67, 70). In addition, Sonic Hedgehog and Indian Hedgehog activates hepatic stellate cells and drive ECM protein synthesis (71). Moreover, hepatocyte YAP and TAZ expression are intimately linked to liver fibrosis and positively correlate with NASH and deleting or silencing TAZ in hepatocytes lowers inflammation and fibrosis in a mouse model of NASH (72–74).

## 3. Concurrent modelling of NASH and atherosclerosis

To investigate the pathophysiology of NAFLD/NASH or ASCVD, multiple well-established animal models have been accepted by the scientific community and are commonly utilized for evaluating translational interpretation of preclinical findings. Below, we will discuss dietary models predominantly administered to mice, highlighting potential limitations of current application when investigating both NAFLD/NASH and ASCVD, as well as non-murine models that may have stronger translational potential but comprise their own set of limitations.

### 3.1. Diets in excess or deficiency: Which is ideal?

Given both NAFLD/NASH and ASCVD arise from dysregulated lipid metabolism and excessive lipid accumulation, the most appropriate models capitalize on genetic and/or dietary lipid loading with additional modifications to exacerbate disease, such as simple carbohydrates or cholesterol. Lipid profiling of mice demonstrates that the majority of their cholesterol is carried in high-density lipoprotein (HDL) particles, contrasting human lipid profile in which HDLs comprise only one-third of total cholesterol (75). Since elevated LDLs and very low-density lipoproteins (VLDLs) are direct contributors to atherogenesis (76), mice will therefore not spontaneously develop atherosclerotic lesions comparable to humans beyond the initial fatty streak (77). Thus genetic (*Ldlr^−/−^* (78), and apolipoprotein E deficient [*Apoe^−/−^*] mice 79, 80) or viral (PCSK9-AAV 81, 82) manipulation is required for mice to develop atherosclerosis. Implementing a combination of genetic dyslipidemia with dietary models to induce NASH permits simultaneous investigation of both NASH and atherosclerosis.

While administration of a high-fat diet in atherosclerotic models is well-established to induce hyperlipidemia and steatohepatitis (83–85), whether high-fat or Western diets are sufficient to elicit all components of NASH (hepatic steatosis, inflammation, and hepatocellular ballooning) and fibrosis remains unclear. Multiple studies report conflicting phenotypes in *Apoe^−/−^* mice following a high-fat diet regimen. For example, Karavia and colleagues demonstrated that despite administration of a high-fat diet (21.2% fat) for 24 weeks, *Apoe^−/−^* mice will accumulate less hepatic triglycerides compared with C57BL/6 mice fed the same diet (86). In contrast, others showed that only 8 weeks of high-fat diet in *Apoe^−/−^* mice was sufficient to induce hepatocellular ballooning and hepatic fibrosis (87). Additional studies by Matsuzawa et al. found that 12–24 weeks of an “atherogenic diet” in C57BL/6J mice is sufficient to induce hepatocellular ballooning and hepatic fibrosis (88), while Zhang et al., induced steatohepatitis with fibrosis and hepatocellular carcinoma following 14 months of high-fat, high-cholesterol feeding in C57BL/6 mice (89). Furthermore, a study by Schierwagen et al. compared Western diet and methionine-choline deficient (MCD)-diet in *Apoe^−/−^* mice, demonstrating significant fibrosis and hepatocellular ballooning in Western diet-fed mice after just 7 weeks (90). Comparisons between diet formulations used in the above studies show that while Karavia and colleagues utilized a Western-type diet, which contains 0.2% cholesterol (86, 91), Schierwagen et al. and Matsuzawa et al. utilized diets containing 1.25% cholesterol (88, 90). Furthermore, Trevaskis and colleagues first described the Amylin liver NASH (AMLN) diet, which contained 2% cholesterol and 40% fat from either partially hydrogenated vegetable oil or lard and induced murine steatohepatitis and fibrosis following 12 weeks feeding (92). Together, these studies highlight the fact that additional components of a high-fat or Western diet, mainly cholesterol, contribute to the NASH phenotype beyond excessive calories from fat (Table 1).

**Table 1.** Murine and non-murine models of NAFLD/NASH with or without atherosclerosis

Disease Model	Animal Model	Diet Source	Diet Components	Time on Diet	Phenotype	References
**Murine models**						
NAFLD	Mouse (C57BL/6J) *Apoe^-/-^*	Mucedola, Milan, Italy	Western-type Diet: 21.2% kCal from fat 0.2% cholesterol	24 weeks	Normal hepatic histology with no triglyceride accumulation noted	Karavia et al. (99)
NAFLD	Mouse (C57BL/6J)	Envigo	Fructose-palmitate diet (TD.160785): 190g/kg hydrogenated vegetable shortening 40g/kg anhydrous milk fat 0.2%–0.5% cholesterol 55% glucose/45% fructose w/w in the drinking water	16 weeks	Enhanced steatosis but no fibrosis	Wang et al. (76)
NASH	Mouse (C57BL/6J) *Apoe^-/-^*	No information provided	High-fat diet No additional information provided	8 weeks	Enhanced plasma AST/ALT, hepatic steatohepatitis, ballooning, and fibrosis	Lu et al. (100)
NASH	Mouse (C57BL/6J)	Oriental Yeast, Tokyo, Japan	Atherogenic diet: 14g fat 1.25% cholesterol High fat diet 60g fat 1.25% cholesterol	12–24 weeks	Enhanced steatosis, inflammation, and fibrosis observed in atherogenic and high fat diet combined, but not atherogenic diet alone	Matsuzawa et al. (101)
NASH	Mouse (C57BL/6J)	Research Diets, New Brunswick, NJ Envigo	High-fat diet (D12492): 60% fat 0.03% cholesterol Choline-deficient high fat diet (D05010402) 60% fat 0.03% cholesterol Choline deficient Western diet (TD.88137): 42% fat, 0.2% cholesterol	15 weeks	Enhanced steatosis. Inflammation only observed in Western diet. Fibrosis only observed in choline deficient high fat diet and Western diet.	Smati et al. (124)
NASH	Mouse (C57BL/6J)	Teklad	Fructose-palmitate-cholesterol (TD.140154): 190g/kg hydrogenated vegetable shortening 40g/kg anhydrous milk fat 1.25% cholesterol ~35% reduction in choline 55% glucose/45% fructose w/w in the drinking water	8–28 weeks	Enhanced steatohepatitis and fibrosis	Wang et al. (74)
NASH	Mouse (C57BL/6J)	Envigo	Fructose-palmitate diet (TD.160785): 190g/kg hydrogenated vegetable shortening 40g/kg anhydrous milk fat 1.25% cholesterol ~35% reduction in choline 55% glucose/45% fructose w/w in the drinking water	16 weeks	Enhanced steatohepatitis and fibrosis	Wang et al. (76)
NASH	Mouse *(Lep*^*ob*^*/Lep*^*ob*^*)*	Research Diets, New Brunswick, NJ	High trans-fat, high fructose, high cholesterol diet (HTF): 40% kCal fat from vegetable shortening 22% w/w fructose 2% cholesterol	12 weeks for trans-fat diet	Enhanced hepatic steatosis and fibrosis	Trevaskis et al. (104)
NASH	Mouse (C57BL/6J)	Research Diets, New Brunswick, NJ	NASH diet (D17010103): 40% kCal fat with 50g/kg primex shortening (non-transfat), 122g/kg corn oil, partially hydrogenated 22% w/w fructose 2% cholesterol	24 weeks	Enhanced AST, ALT, and ALP, with enhanced steatohepatitis and fibrosis	Rom et al. (96, 97)
NASH-HCC	Mouse (C57BL/6J)	Specialty Feeds, Glenn Forrest, WA	High-fat/high-cholesterol diet: 43.7% fat 0.203% cholesterol	8–14 months	Enhanced AST, ALT, steatohepatitis, and fibrosis beginning at 8 months, and HCC observed by 10 months	Zhang et al. (102)
NASH and Atherosclerosis	Mouse (C56BL/6J) *Ldlr*^*-/-*^*.Leiden*	Research Diets, New Brunswick, NJ	High-fat diet (D12451): 45% kCal fat from lard 35% kCal carbohydrates from sucrose 0.01% w/w cholesterol Fast food diet: 41% kCal fat from milk fat 44% kcal carbohydrates from fructose 0.05% w/w cholesterol	28 weeks	Enhanced AST, ALT, hepatic steatohepatitis, fibrosis, and atherosclerosis	Van den Hoek et al. (86)
**Non-murine models**						
Coronary artery disease	Ossabaw pig	No information provided	Western diet: 38% kCal fat 1.5% cholesterol w/w	6 months	Significant increases in ALP, ALT, AST (but no liver histology noted), and coronary artery lesions compared with control diet	Matthan et al. (133)
NASH	Ossabaw pig	Purina TestDiet, Inc., Richmond, IN	Atherogenic diet: 46% kCal fat 20% kCal fructose 2% cholesterol 900 ppm choline Modified atherogenic diet: 43% kCal fat 17.8% fructose 2% cholesterol 700ppm choline	24 weeks	Enhanced AST and ALT, steatohepatitis, ballooning, and fibrosis in modified atherogenic diet group. Enhanced ALT and steatosis in atherogenic diet group.	Lee et al. (134)
NASH with fibrosis by NaNO_2_ injections	Wistar rat	Research Diets, New Brunswick, NJ	Choline deficient high fat diet (A06071302): 60% kcal fat 0.03% cholesterol 0.1% methionine Choline deficient NaNO_2_ injections: 10-30mg/kg	10 weeks, with NaNO_2_ administered following 4 weeks	Enhanced fibrosis	Schwabl et al. (126)
NASH and atherosclerosis	Japanese white rabbit	No information provided	High-fat and -cholesterol diet: 12% corn oil 0.75% cholesterol	9 months	Enhanced steatohepatitis, hepatic fibrosis, and aortic atheroma	Ogawa et al. (135)
NASH and atherosclerosis by aortic endothelial injury by balloon catheter	Rabbit	TestDiet, Saint Louis, MO	Cholesterol containing chow diet: 2.4% fat w/w 1% cholesterol	3 months	Enhanced hepatocyte ballooning and fibrosis. Atherosclerosis only enhanced with cholesterol diet and injury	Taylor et al. (139)
NASH	Cynomologus monkey	Kunming Biomed International	High fat diet: 20% fat, 5% cholesterol	3 years	Enhanced steatohepatitis, fibrosis, and NAS score	Lyu et al. (142)
NASH	Cynomologus monkey	Beijing Keao Xieli Feed Co., Ltd, Beijing, China	High fat high cholesterol diet: 10% lard 15% cholesterol	24 weeks	Enhanced steatohepatitis, hepatic ballooning, fibrosis, and NAS score	Jian et al. (143)
NASH	Cynomologus monkey	Keao Xieli	High fat high cholesterol diet: 10% lard 1% cholesterol	16 weeks	Enhanced NAS score, steatosis, and fibrosis	Zang et al. (144)

Supplementation of a high-fat diet with cholesterol appears to be a major contributor to the pathogenesis of NASH. Analysis of liver biopsies from patients with NASH demonstrated that free cholesterol accumulation associates with hepatic steatosis and continues to increase with the progression of NASH (93). In addition, unlike triglycerides or free fatty acids, cholesterol loading is sufficient to deplete mitochondrial glutathione in hepatocytes resulting in sensitivity to inflammatory cytokines (94). Following extended high-fat, high-cholesterol feeding for 14 months, cholesterol induces gut microbiota dysbiosis, enhanced gut leakiness, endotoxemia, and bile acid biosynthesis in C57BL/6 mice, which result in NASH with fibrosis and HCC (89). However, the effects of dietary cholesterol and the risk of CVD remains unclear (95). Since conventional atherogenic diets parallel human consumption of cholesterol (96, 97), but typically contain approximately one-tenth that of murine NASH diets (0.2% and 2%, respectively (83, 92)), excessive cholesterol supplementation may be inappropriate for the comparative studies of CVD and NASH together. Thus, other components such as dietary sugars may be considered when addressing models for concurrent NASH and ASCVD.

Since fructose largely replaced sucrose as a source of sweeteners in soft drinks in the 1970's, an association between high-fructose corn syrup consumption and obesity became increasingly observed (98). In addition, beyond increasing hepatic steatosis, fructose enhances aortic wall thickness and foam cell count in Sprague-Dawley rats fed a high-fat diet (99). Van den Hoek and colleagues fed *Ldlr^−/−^*.Leiden mice an obesogenic diet for 28 weeks containing 41% calories from fat, 0.05% cholesterol, and 44% calories from fructose (100), which recapitulated multiple aspects of NASH like inflammation (100), fibrosis (101), and circulating AST and ALT (102), as well as established atherosclerotic lesions (100). Since *Ldlr^−/−^*.Leiden mice are susceptible to diet-induced obesity and metabolic syndrome compared with conventional *Ldlr^−/−^* mice (103), this model proved effective in examining both fibrotic NASH and atherosclerosis (100). While normal consumption of fructose feeds into glycogen biosynthesis (104), excessive fructose consumption suppresses fatty acid β-oxidation (FAO) in the liver (105) and induces DNL by the induction of sterol regulatory element-binding protein-1 (SREBP1), acetyl-CoA carboxylase-1 (ACC1), and fatty acid synthase (FAS) (105, 106). By comparing the supplementation of fructose to glucose in humans and mice, Stanhope et al. and Softic et al. demonstrated that inhibition of FAO and induction of DNL are caused specifically by high intake of fructose, and not glucose (107–109). In the gastrointestinal tract, fructose deteriorates the gut barrier and promotes chronic inflammation by endotoxemia (110). Since endotoxemia is associated with liver disease and atherosclerosis (111, 112), the effects of fructose on the development of NAFLD/NASH and ASCVD may be due to chronic inflammation secondary to enhanced gastrointestinal permeability. Thus, the contribution of high-fructose intake for the concurrent development on NASH and atherosclerosis warrants further research.

Although diets with excess nutrients elicit NASH or ASCVD pathology, diets lacking key nutrients are an additional avenue for inducing disease. Choline and methionine deficiency diminishes VLDL assembly and reduces triglyceride clearance but results in weight loss (113), contrasting with typical weight gain associated with most human NASH. The MCD diet was previously viewed as a conventional NASH model; however, multiple groups demonstrated that MCD does not cause insulin resistance (114) and enhances weight loss despite hepatic steatosis (115), highlighting the disconnect between human disease characteristics and disease in MCD diet-fed mice. Since the MCD model clearly has its deficiencies in application with NASH pathology, researchers have developed modifications of this model to align the diet-induced phenotype more closely with human NASH. For example, the high-fat, choline-deficient diet induces steatosis, inflammation, and fibrosis over a 15-week period; however, it does not induce ballooning (116). Choline deficiency reduces pro-atherogenic VLDL assembly (113) but choline supplementation has no effect on atherosclerotic plaque area (117). The choline-deficient high-fat diet with no added choline but 0.1% methionine has approximately 0.03% cholesterol and induces steatohepatitis (116); however, to develop fibrosis the addition of 25 mg/kg NaNO_2_ (118) is required to induce hypoxemia (119). Enhancing methionine to 0.2% does prevent weight loss while enhancing NASH and hepatic fibrosis (113). While enhancing liver fat accumulation, the choline-deficient high-fat diet actually attenuates fasting plasma insulin and improves glucose tolerance (120). Patients with NAFLD develop hyperinsulinemia as a result of impaired whole-body insulin clearance, which may further drive hepatic steatosis (121). The positive correlation between hyperinsulinemia and atherosclerosis has been long-established (122). Therefore, models mimicking hyperinsulinemia, should be considered in appropriate models of both NASH and ASCVD.

### 3.2. Non-murine models

The utilization of mice for pathological modelling has its benefits. For example, mice gestate and grow rapidly, require small spaces for housing, are relatively inexpensive to care for, and are easily genetically manipulated. While numerous mouse models have been implemented to study NAFLD/NASH or ASCVD, each provides a unique set of limitations. For example, atheroprone mice must first be “humanized” with genetic manipulation to shift their endogenous plasma cholesterol composition. Furthermore, as outlined in section 2.1, many mouse models of NASH do not completely mimic all aspects of the human disease, particularly hepatocellular ballooning and fibrosis (123). Additionally, dietary models alone are insufficient to induce atherosclerosis in mice due to their plasma lipid composition (77). Therefore, the use of non-murine or large animal models that spontaneously develop atherosclerosis may provide a more accurate representation of both human NASH and ASCVD.

Porcine models of atherosclerosis are closely related to the human disease due to similar lipoprotein composition; thus, pigs do not require genetic modification to induce ASCVD (124). In addition to their use as an atherogenic model (125), miniature Ossabaw pigs develop metabolic syndrome with abnormal liver pathology indicative of NASH when fed a modified high-fat, low-choline diet for 24 weeks (126). However, pigs require larger housing facilities, utilize more resources, and are therefore not as cost-effective. Rabbits may be a useful alternative to pigs or mice because they require less resources than pigs and are able to develop NASH with fibrosis following 9 months of a modified diet containing 0.75% cholesterol and 12% corn oil (127). Rabbits were pivotal in the initial discovery of atherosclerosis in which the Russian physician Ignatowski observed aortic plaques in rabbits fed an enriched animal fat and protein diet (128). Since then, rabbits are widely used for atherosclerosis studies due to their similarities to human lipoproteins, and both diet-and genetically-induced atherosclerotic models have been implemented (129). Furthermore, 1% cholesterol-fed rabbits develop both atherosclerosis (130) and fibrotic NASH, representing a simple model to investigate both diseases simultaneously (131). However, rabbits show wide genetic variability compared with mice (129) and therefore require larger cohorts to observe meaningful differences between treatment groups. Perhaps the most translatable model of either NASH or atherosclerosis is the use of nonhuman primates. For example, cholesterol metabolism between humans and Baboons is remarkably similar (132), and baboons given a high-sugar, high-fat diet leads to weight gain and hyperlipidemia similar to humans (133). Cynomolgus monkeys given a diet containing 20% fat with 5% cholesterol developed NASH with fibrosis (134). In addition, a high-fat, high-cholesterol (1%) diet can accelerate NASH in Cynomolgus monkeys with spontaneously-developed NASH symptoms (135, 136). However, the ethical considerations of these animals should be heavily weighed when deciding which models are the most appropriate. Despite their obvious similarities with humans, the advanced cognition of nonhuman primates sheds light on the moral obligations of scientific researchers (137).

## 4. Emerging biomarkers linking NAFLD/NASH and ASCVD offer potential therapeutic strategies

**Figure 2:**
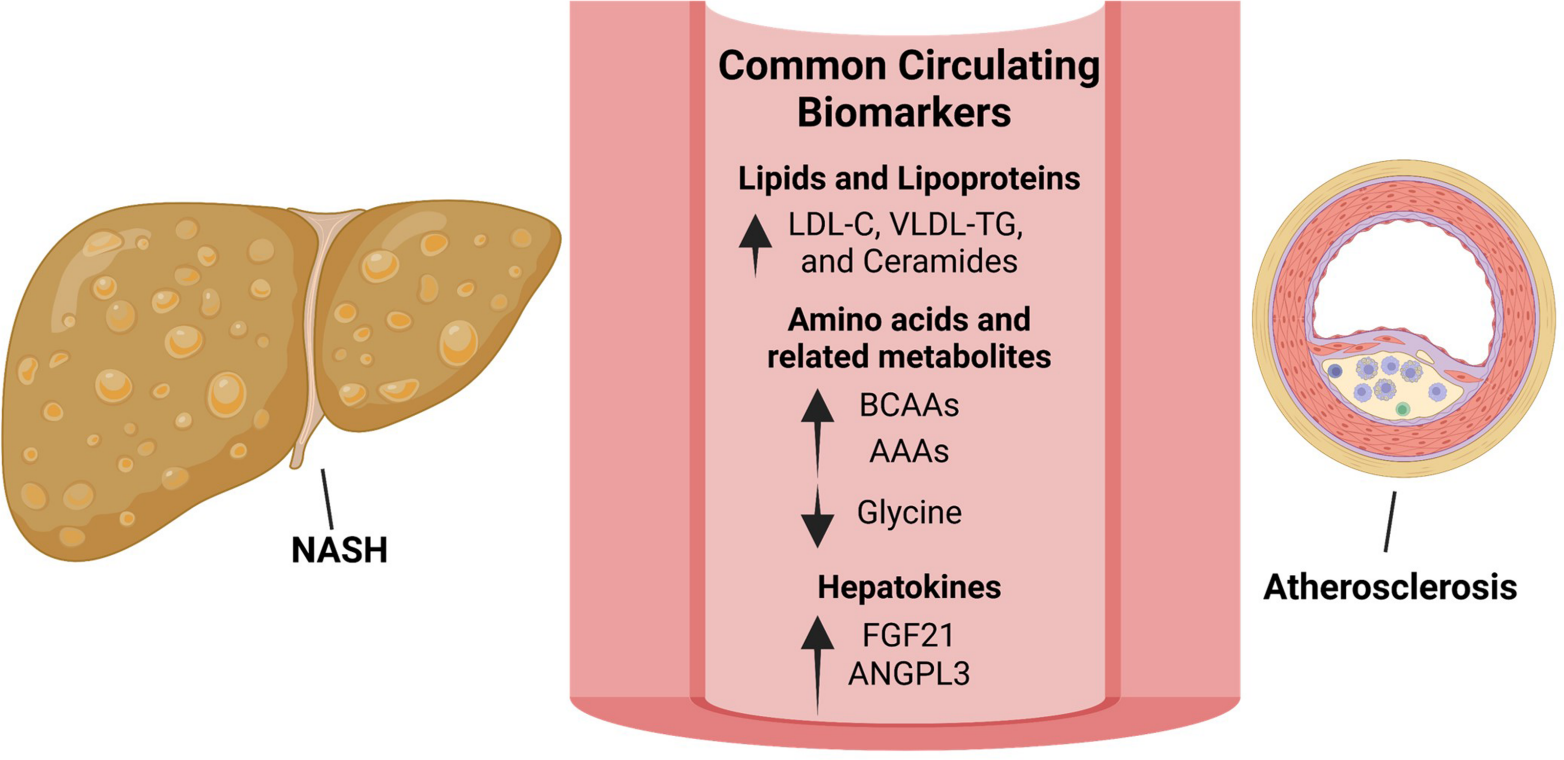
Biomarkers linking NAFLD/NASH and ASCVD offer potential therapeutic strategies. Lipid species that are increased in both NAFLD/NASH and ASCVD include low-density lipoprotein-cholesterol (LDL-C), very low-density lipoprotein (VLDL) and triglycerides (TG) as well as ceramides. Although significantly lowering ASCVD, LDL-C reduction using statins has shown inconsistent results with regards to NASH treatment. Targeting ceramide synthesis have shown promising results in rodent models of NAFLD/NASH and ASCVD and warrants clinical evaluation. Amino acid metabolism is commonly dysregulated in NAFLD/NASH and ASCVD with circulating branched-chain amino acids (BCAAs) and aromatic amino acids (AAAs) increased and glycine decreased in both diseases. In rodent models and small-scale clinical studies, glycine-based treatments reduced steatohepatitis and atherosclerosis warranting clinical evaluation in larger cohorts. The hepatokines, fibroblast growth factor-21 (FGF-21) and angiopoietin like 3 (ANGPTL3), are increased in both NAFLD/NASH and ASCVD. Interestingly, approaches to inhibit hepatic ANGPTL3 have shown promise in treating dyslipidemia but were associated with increased hepatic steatosis and markers of liver injury. Despite being increased in both diseases, FGF21 analogues are protective in rodent models of NASH and atherosclerosis as well as in patients with NASH.

The circulating levels of liver enzymes (aspartate transaminase [AST], and alanine transaminases [ALT]), other nonenzymatic proteins (albumin) and metabolites of heme (bilirubin) are routinely used to diagnose and monitor liver diseases, including NAFLD/NASH (138). While liver function tests are routinely preformed, their interpretation is often challenging and their relevance to CVD, the main cause of death in patients with NASH (7–12, 139–141) is limited. Furthermore, predictive biomarkers of NASH are lacking, resulting in invasive biopsy as the only method for diagnosis (51). Established biomarkers for CVD including C-reactive protein (CRP), cardiac troponins I and T, B-type natriuretic peptides, and D-dimer, are widely used for diagnosis and management of various CVDs including atherosclerosis, myocardial infarction, acute coronary syndrome, cardiac arrest, thrombosis, and ischemic cardiac diseases (142–144). Despite the wide use of these biomarkers for diagnosis and monitoring, there remains a need to identify new pathological pathways and pertinent biomarkers that can be useful for concurrent diagnosis and monitoring of NAFLD and CVD. Herein, we explore established and newly identified biomarkers that are closely related to NAFLD/NASH and ASCVD (Figure 2).

### 4.1. Lipids, lipoproteins, and lipid peroxidation products

The liver is the major site of lipid and lipoprotein metabolism and regulates the production and clearance of all classes of lipoprotein particles (145). In addition, the liver regulates the metabolism of the major lipoprotein components including triglycerides and cholesterol (146, 147). Dysregulation of hepatic lipid metabolism leading to excess lipid accumulation is a hallmark feature of NAFLD which further promotes atherogenic dyslipidemia and the risk of ASCVD (147, 148). Thus, alteration in circulating lipoproteins in patients with NAFLD is considered an early biomarker to predict the risk of ASCVD. Preclinical and clinical reports showed that improvement in NAFLD improves dyslipidemia (149–151); however, statins or other lipid-lowering agents did not reduce the risk of cardiovascular mortality in patients with NAFLD (152). In contrast, pemafibrate, a PPARα modulator that lowers triglycerides, VLDL, and cholesterol, did not reduce the incidence of cardiovascular events but lowered the incidence of NAFLD (153). These studies highlight the need to improve our understanding of the role of other lipid and non-lipid metabolites, not only as biomarkers linking these two diseases, but also as potential targets for concurrent therapy.

Enhanced influx of free fatty acids to the liver, oxidative stress and inflammatory stimuli promotes the synthesis of hepatic ceramides in NAFLD (154, 155). Ceramides are active lipid intermediates of the sphingolipid family that are produced mainly in the liver (156). Beyond their increased levels in the liver, circulating ceramides are elevated in animal models and patients with NAFLD (157, 158), particularly in those with NASH (159), where they are found mainly in VLDL and LDL particles (159, 160). Moreover, various ceramide species (mainly Cer16:0, Cer18:0 and Cer24:1) are consistently associated with adverse cardiovascular outcomes and mortality (161–163), and have been suggested as biomarkers for ASCVD beyond the currently exciting risk factors (164). Ceramides are not only associated with ASCVD but can also increase atherosclerosis by promoting endothelial dysfunction (154, 165). Pharmacological (myriocin) and genetic (hepatic deletion of dihydroceramide desaturase-1) approaches targeting ceramide synthesis not only lowered hepatic steatosis and fibrosis (166, 167), but also reversed endothelial dysfunction and atherosclerosis in rodent models (168, 169).

In NAFLD, and particularly in NASH, hepatic mitochondrial dysfunction augments ROS production promoting lipid peroxidation, the oxidation of polyunsaturated fatty acids *via* lipid-peroxyl radical reaction (54, 170–172). Beyond enhanced hepatic lipid peroxidation, an increase in systemic markers of lipid peroxidation (e.g., malonaldehyde, MDA) is well-documented in both experimental models and in patients with NAFLD (173–175). Furthermore, higher circulating MDA in patients with NAFLD is associated with lower antioxidant capacity of HDL and subclinical atherosclerosis (176). Peroxidation of lipoproteins (mainly ox-LDL) plays critical roles in various steps of atherosclerosis development (177), including endothelial activation and dysfunction (178, 179), monocyte adhesion (180, 181), macrophage-foam cell formation (182–185), and proliferation and migration of vSMCs (186, 187). Indeed, circulating ox-LDL is a useful marker in predicting the risk of coronary artery diseases (CAD) (188) as well as NAFLD severity (189). In addition, circulating ox-LDL in the form of MDA-LDL is not only increased in individuals with NAFLD, but is also associated with high-risk atherosclerotic plaques in the same patients (190).

### 4.2. Amino acids

While dysregulated lipid metabolism in NAFLD/NASH and ASCVD has been extensively studied, recent evidence strongly suggests altered amino acid metabolism as a common factor in both diseases (83, 191–203). Gaggini et al. (192) found that most circulating amino acids were elevated among obese subjects with NAFLD and further increased in the presence of insulin resistance (IR) and obesity. Patients with more advanced liver damage and fibrosis had higher levels of the branched-chain amino acids (BCAAs, leucine, isoleucine and valine) (204) and aromatic amino acids (AAAs tryptophan, phenylalanine, and tyrosine) (192, 205). Furthermore, BCAAs and AAAs are consistently reported to be positively associated with increased risk for ASCVD independent of hypertension and metabolic disease (206–209).

Despite the associations between elevated circulating amino acids and NAFLD or ASCVD, a causative role of BCAAs and AAAs remains unclear. In mice with NAFLD, BCAAs promote liver injury and apoptosis by downregulating lipid-induced autophagy (210). In contrast, BCAA supplementation to mice fed high-fat or choline-deficient, high-fat diets lowered hepatic steatosis and injury through suppression of hepatic lipogenic genes and modulation of intestinal microbiota-mediated production of acetic acid (211, 212). These contrasting effects may be due to specific BCAAs, since the adverse metabolic effects in obese mice appear to be mediated by isoleucine and valine but not by leucine, whose restriction aggravated hepatic steatosis (213). In addition, leucine protects against macrophage foam cell formation by inhibiting lipid biosynthesis, promoting cholesterol efflux and enhancing mitochondrial respiration (191, 197, 214, 215). Furthermore, *Apoe*^−/−^ mice supplemented with leucine showed enhanced hepatic cholesterol efflux, which effectively reduced circulating LDL and atherosclerosis (216). The effects of BCAAs on different cell types may differentially regulate the pathogenesis of atherosclerosis. For example, supraphysiological levels of BCAAs (6 mmol/L) enhanced ROS and activated endothelial cells (217). In contrast, physiological levels of leucine (0.2 mmol/L) protect against macrophage foam cell formation by inhibiting lipid biosynthesis, promoting cholesterol efflux and enhancing mitochondrial respiration (191, 197, 214, 215). Thus, future studies are warranted to clarify the causative role of exogenous BCAAs and determine the effects of individual BCAAs in NAFLD/NASH and ASCVD.

In individuals with histologically confirmed NAFLD, plasma phenylalanine was increased only in those with NASH, while tyrosine was increased in both patients with simple steatosis and NASH (218). Tyrosine and total AAAs were associated with NAFLD severity assessed by hepatocellular ballooning, inflammation and fibrosis in patients with NASH (192, 205). Also, serum AAAs were reported to be higher in patients with NASH, but when compared to patients with simple steatosis, only tryptophan was higher in those with NASH. In addition, serum tryptophan and tyrosine were positively correlated with total and LDL-cholesterol (219), suggesting that alterations in circulating AAAs are associated with the risk of NAFLD-associated CVD. Indeed, in a large cohort of adults Finns, circulating tyrosine was positively associated with subclinical atherosclerosis assessed by carotid intima-media thickness (IMT) (200). In addition, phenylalanine and tyrosine were associated with CAD, ischemic stroke, and cardiovascular events (220). While the above studies demonstrate increased circulating AAAs in NAFLD/NASH and ASCVD, studies addressing the causative role of altered AAA metabolism and the effects of individual AAAs in the development of these diseases are lacking.

Whereas circulating BCAAs and AAAs are increased, glycine, the simplest amino acid, is consistently reported to be lower in association with suppressed hepatic glycine biosynthetic genes (e.g., alanine-glyoxylate aminotransferase [*AGXT*] and serine hydroxymethyltransferase [*SHMT*]) and inversely associated with the risk or severity of NAFLD/NASH, CVD and related cardiometabolic diseases in both mouse models and patients (83, 192, 195, 196, 199, 201, 221–225). While these reports highlight lower circulating glycine as an emerging biomarker for both NAFLD/NASH and ASCVD, studies in humans and mice support a causative role of reduced glycine availability and the potential of glycine-based treatment in both diseases (83, 199, 201). Glycine is a nonessential amino acid mainly synthesized in the liver (226). In patients and mice with NAFLD, glycine is a limiting substrate for *de novo* synthesis of glutathione (GSH), the most abundant endogenous antioxidant (83, 199). Therefore, the decrease in circulating glycine in NAFLD may be explained by insufficient hepatic production coupled with enhanced demand for GSH biosynthesis. Furthermore, glycine restriction aggravates atherosclerosis in *Apoe^−/−^* mice (83, 195). Glycine or glycine-based treatments [e.g., serine, trimethylglycine (betaine) and a glycine-based tripeptide, DT-109] lowered hepatic steatosis, inflammation and fibrosis as well as atherosclerosis in various rodent models (83, 195, 227) and humans (199) through mechanisms involving hepatic GSH biosynthesis, enhanced fatty acid utilization, suppression of proinflammatory/fibrotic responses and modulation of the gut microbiome. In addition to glycine, glutamate, another component of GSH, is increased in NAFLD/NASH, which has been attributed to gamma-glutamyltransferase-mediated glutamate release during GSH transamination and upregulation of hepatic glutaminase-1 (192, 203). This, together with alternations is serine metabolism in NAFLD (192, 198), serve as a basis for the glutamate-serine-glycine (GSG) index, which recently emerged as a potential biomarker for the severity of NAFLD and fibrosis (192, 228).

### 4.3. Polyamines

Polyamines including putrescine, spermidine, and spermine are present in all living organisms. These aliphatic polycation compounds play a role in various biological events including maintenance of chromatin structure, gene transcription and translation, cell growth, and proliferation. The biological effects of polyamines are believed to be mediated by modulation of protein-protein and protein-DNA interactions (229–231). Emerging evidence suggests that polyamines modulate the risk of CVD, metabolic diseases, neurological disorders, and cancer (232–235). Nevertheless, the role of polyamine metabolism as a potential link between NAFLD/NASH and CVD remains to be explored.

Dysregulated metabolism of polyamines in NASH has been identified in human and rodent studies. A metabolomics-based study demonstrated that circulating spermidine was more than 2-fold lower in individuals with advanced NASH and fibrosis compared to those with the early disease (236). Alternations in polyamine metabolism during NASH could be attributed to the availability of S-adenosylmethionine (SAMe), a universal methyl donor and a polyamine precursor. In NASH, glycine-N-methyl transferase (GNMT), which catalyzes the transfer of a methyl group from SAMe to glycine, is reduced, promoting an increase in SAMe and subsequent accumulation of putrescine associated with enhanced lipid peroxidation (237). While changes in circulating putrescine in NAFLD/NASH have not been reported yet and the evidence for decreased spermidine is limited (236), a number of studies reported a protective role of spermidine in mouse models of NAFLD. In diet-induced obese mice, supplementation with spermidine lowered hepatic steatosis associated with downregulation of lipogenic genes and upregulation of genes driving FAO, including *Ppara* (233, 238). Also, spermidine ameliorated obesity-associated NAFLD in mice by increasing the phosphorylation of hepatic AMP-activated protein kinase (AMPK), which in turn inhibited the expression of the lipogenic genes *Srebf1c* and *Fas* (239). In addition, spermidine treatment restored the hypusination of translation factor EIF5A, which was decreased in NASH, leading to enhanced mitochondrial FAO and protection against diet-induced NASH in mice (240). While the studies above suggest dysregulated polyamine metabolism in NASH and indicate a protective role of spermidine, further research is needed to establish the use of polyamines as biomarkers for NAFLD/NASH.

With regards to CVD, the association with spermidine has been evaluated in a number of recent studies. In individuals with AMI, serum spermidine was associated with improved prognosis and reduced rates of major adverse cardiac events (241). On the other hand, a higher risk of stroke was found with an increasing baseline serum spermidine (242). Moreover, obese and overweight subjects were found to have higher serum spermidine along with increased atherogenic markers including triglycerides, total and LDL-cholesterol (243). While the above association studies appear to be conflicting, intervention studies in mouse models consistently demonstrated athero/cardioprotective properties of spermidine. In *Apoe*^−/−^ mice, spermidine supplementation lowered plaque lipid accumulation and necrotic cores. Spermidine triggered cholesterol efflux in autophagy-competent but not in autophagy-deficient VSMCs or macrophages lacking autophagy related 7 (*Atg7*) (244). In addition, spermidine and spermine protected against LDL oxidation resulting in reduced uptake of ox-LDL by macrophages (245). Furthermore, spermidine decreased cardiac hypertrophy and preserved diastolic function in old mice concomitant with enhanced cardiac autophagy, mitophagy and mitochondrial respiration. These cardioprotective effects were abolished in mice lacking *Atg5* in cardiomyocytes (232), supporting the notion that induction of autophagy by spermidine may be useful to prevent CVD. Interestingly, in humans, higher consumption of dietary spermidine was associated with lower CVD incidence (232). Together, while spermidine supplementation appears to be protective against NAFLD/NASH and CVD in mouse models, the use of spermidine or other polyamines as biomarkers and the therapeutic potential of spermidine in clinical settings warrant further research.

### 4.4. Oxalate

Oxalate is the ionic form of oxalic acid, and is an end-product of glyoxylate metabolism in the liver, which accounts for 80%–90% of total circulating oxalate (246–248). The vast majority of oxalate (>90%) is eliminated through the kidneys (249, 250). Although humans have no enzymes capable of degrading oxalate (251), specific hepatic enzymes can prevent oxalate overproduction *via* the detoxification of glyoxylate to glycolate (by glycolate reductase/hydroxypyruvate reductase, GRHPR) or glycine (by AGXT) (252). Genetic defects in these enzymes result in primary hyperoxaluria, in which toxic levels of oxalate are produced by the liver (252). Furthermore, increased systemic oxalate can also be caused by impaired oxalate excretion in chronic kidney disease (250, 253). Beyond this, increased serum or urine oxalate has recently been linked with NAFLD/NASH (83, 248) and CVD (83, 248, 253, 254).

Suppression of glyoxylate detoxifying genes, particularly *AGXT*, has been consistently reported in both in humans and mice with NAFLD/NASH. Assessment of hepatic gene expression in patients who had undergone bariatric surgery revealed that *AGXT* is downregulated in those with NASH (255). In support, *AGXT* and *GRHPR* were recently reported to be downregulated in steatotic hepatocytes isolated from patients with NAFLD (248). Analysis of liver transcriptomic data from several cohorts of patients with various degrees of liver disease (steatosis, NASH, cirrhosis, and HCC) combined with data from mice with NAFLD or NASH revealed that *AGXT* was consistently downregulated in all human and mouse cohorts (83, 196, 248, 256). Furthermore, aggravated NASH and fibrosis in *Agxt*^−/−^ mice fed a NASH-inducing diet suggest a causative role of oxalate in NAFLD (83). Nevertheless, future studies evaluating the liver and circulating levels of oxalate in patients with NAFLD/NASH are warranted.

With regards to CVD, increased circulating oxalate has been associated with increased cardiovascular morbidity and mortality. Among hemodialysis patients, serum oxalate was positively associated with cardiovascular risk factors including elevated pulse wave velocity, central aortic systolic and diastolic blood pressures, and risk for cardiovascular events (253, 257). In patients with end-stage renal disease, increased circulating oxalate was not only associated with CVD events, but also with aggravated dyslipidemia (increased triglycerides and VLDL-cholesterol, and decreased HDL-cholesterol) and proatherogenic cytokines and chemokines (IL-6, TNFα, and monocyte chemoattractant protein-1) (254). In patients with significant CAD and normal kidney function, and in atherosclerotic *Apoe*^−/−^ mice, we found a significant decrease in the glycine to oxalate ratio aligned with downregulated hepatic AGXT. In mice deficient with both *Agxt* and *Apoe*, as well as in *Apoe*^−/−^ mice challenged with exogenous oxalate, atherosclerosis was increased with enhanced superoxide and CCL5 in atherosclerotic lesions. These effects were reversed by AAV-mediated overexpression of AGXT in livers of *Apoe*^−/−^ mice, indicating a causative role of oxalate overproduction in atherosclerosis (196). At the cellular level, oxalate was reported to induce mitochondrial dysfunction, oxidative stress and the release of proinflammatory chemokines and cytokines in endothelial cells, monocytes, and macrophages (196, 258–260). Together, the association between circulating oxalate, NAFLD/NASH and ASCVD should be further studied in larger cohorts including patients without kidney disease.

### 4.5. Hepatokines

The liver secretes various proteins known as hepatokines that can regulate systemic metabolic homeostasis through a crosstalk with other organs including skeletal muscle, adipose tissue, the central nervous system and blood vessels (261). In addition to their metabolic role, systemic alterations in hepatokines are implicated in several pathological conditions including IR, diabetes and CVD (261, 262); however, evidence regarding the role of hepatokines as modulators of atherosclerosis is limited.

Angiopoietin-like 3 (ANGPTL3) is a glycoprotein that is expressed and secreted primarily by the liver (263). Secreted ANGPTL3 binds lipoprotein lipase and inhibits its activity to hydrolyze lipoprotein triglycerides into fatty acids that are taken up by metabolic tissues. As a result, circulating triglycerides are increased (264, 265). Indeed, individuals with loss-of-function mutations in *ANGPTL3* have lower plasma triglycerides (266). In a cross-sectional investigation of obese subjects, both hepatic and plasma ANGPTL3 were higher in individuals with NALFD and positively correlated with hepatic steatosis and histological markers of NASH (267). Among patients with various degrees of NAFLD, serum ANGPTL3 was increased in individuals with NASH, but not in those with simple steatosis (268). With regards to CVD, a study involving 1,493 MI cases and 3,231 controls demonstrated that individuals with lower plasma ANGPTL3 had a reduced risk of MI (269). In line, increased plasma ANGPTL3 was positively associated with the severity of coronary stenosis among patients with angina (270). Beyond its potential as a biomarker, the efficacy of ANGPTL3 inhibition has been studied extensively in preclinical and clinical settings. Both in *Ldlr*^−/−^ mice treated with antisense oligonucleotides (ASO) targeting *Angptl3* and in APOE*3Leiden.CETP mice treated with an antibody against ANGPTL3 (evinacumab), hypercholesterolemia, hypertriglyceridemia and atherosclerosis were significantly decreased (271, 272). Evinacumab also lowered fasting triglycerides and LDL-cholesterol in a phase I trial (271). In a phase IIb trial, administration of vupanorsen, an ASO targeting hepatic *ANGPTL3*, to patients with hypercholesterolemia and hypertriglyceridemia significantly reduced triglycerides together with a modest decrease in LDL-cholesterol. Unfortunately, at higher doses, vupanorsen administration was associated with increased hepatic fat, and over 3-fold elevations in ALT and AST (273). These studies highlight the potential complications in determining dosage for therapeutics like vupanorsen.

Fibroblast growth factor-21 (FGF-21) is a hormone primarily produced and secreted by the liver (274, 275). The hepatic expression and circulating levels of FGF21 are consistency reported to be higher in NAFLD, and are associated with enhanced hepatic necroinflammation and fibrosis (276–280). Furthermore, FGF21 was positively correlated with total cholesterol and triglycerides, and multivariate regression analysis indicated that FGF21 is an independent risk factor of CAD (281). Moreover, serum FGF21 predicted the incident of ASCVD events independent of NAFLD and other traditional cardiovascular risk factors (282, 283). Despite these findings indicating elevated circulating FGF21 as a common biomarker for NAFLD and ASCVD, FGF21 is known for its protective properties in both diseases. An extensive body of literature have demonstrated the protective effects of recombinant FGF21 or FGF21 analogues in preclinical models of NASH (284, 285) and atherosclerosis (286, 287) as well as in patients with NASH (288, 289), serving as an attractive therapeutic marker for both diseases.

Fetuin-A, also known as α2-Heremans-Schmid glycoprotein (AHSG), is synthesized and secreted predominantly by the liver and is among the first hepatokines identified to regulate metabolism through multiorgan crosstalk (290–292). Elevated fetuin-A levels are positively correlated with liver fat, patients with NAFLD, IR, and hepatic fibrosis (293–295). The link between fetuin-A, NAFLD and other metabolic disorders has sparked interest in its involvement in CVD; however, these studies yielded inconsistent results. In a case-cohort investigation, higher circulating fetuin-A was associated with MI and ischemic stroke after adjustment for confounders (296). In contrast, lower plasma fetuin-A, independent of traditional CVD risk factors, was found to be associated with increased CVD mortality among 1,620 patients with CAD (297). Therefore, while fetuin-A may serve as a potential biomarker in NAFLD, the conflicting findings above indicate that fetuin-A may not be a useful biomarker in ASCVD.

## 5. Dual-targeting of NASH and ASCVD: Limitations, caveats, and potential directions

Significant advances in our understanding of the mechanisms that drive NASH have led to the development of numerous of drug candidates that target different pathways in the pathogenesis of NASH. As extensively reviewed (298, 299), these candidates include drugs that target insulin/glucose homeostasis, lipid metabolism, proinflammatory/profibrotic responses, and the gut-liver axis, alongside pharmacological/surgical approaches aimed at lowering body weight. A limited number of drugs that demonstrated efficacy in phase IIb trials were or are currently evaluated in phase III trials. A few drugs approved for other metabolic diseases (e.g., T2D, and obesity) are evaluated as potential treatments for NAFLD/NASH in phase IV trials. While the current therapeutic pipeline in NASH (298, 299) and emerging approaches to treat ASCVD *via* modifying inflammation (300) have been comprehensively reviewed, in this section we discuss (1) potential cardiovascular consequences of promising drug candidates for NASH, and (2) the effects of commonly used (lipid-lowering) and new (anti-inflammatory) drugs for ASCVD on NASH.

### 5.1. Antidiabetic drugs for concurrent treatment of NASH and ASCVD

The prevalence of NAFLD and NASH in patients with T2D is higher than the general population and was estimated at 55% and 37%, respectively (5). As T2D is closely associated with NASH, a number of antidiabetic drugs have been considered as potential treatments for NASH. Among these drugs, pioglitazone, a PPARγ agonist and insulin sensitizer, is currently evaluated in a phase IV clinical trial for NASH (NCT00994682). Pioglitazone administered for 18 months to prediabetic or T2D patients with biopsy-proven NASH effectively lowered NAS and fibrosis scores while improving insulin sensitivity (300–302). However, pioglitazone treatment was associated with weight gain compared to placebo (302). Moreover, pioglitazone was associated with other adverse effects including enhanced risk of hospitalization for heart failure due to fluid retention (303–305). Despite this, accumulating evidence suggests a protective effect of pioglitazone on atherosclerosis-driven events including MI and ischemic stroke. In patients with impaired glucose tolerance or T2D, pioglitazone reduced carotid IMT (306, 307) and atherosclerotic plaque inflammation in association with decreased CRP and increased HDL-cholesterol (308, 309). Furthermore, pioglitazone treatment was associated with reduced total and LDL-cholesterol, triglycerides, and lipoprotein (a) (310–312). Therefore, the cardiovascular consequences of pioglitazone in patients with NASH warrant further research in long-term, large clinical trials.

Newer antidiabetic drug classes, including glucagon-like peptide 1 (GLP1) receptor agonists and sodium-glucose cotransporter-2 (SGLT2) inhibitors, have emerged as potential therapies for NASH. GLP1, an incretin secreted from intestinal L-cells, enhances glucose-stimulated insulin secretion and promotes satiety (313–316). Liraglutide is a GLP1 analogue known to lower body weight (317). In a phase II trial including overweight patients with biopsy-confirmed NASH, 48 weeks of liraglutide treatment was associated with higher rates of NASH resolution and attenuation of fibrosis (318). Stable isotope studies in patients treated with liraglutide, supported by lipid flux studies in human primary hepatocytes, demonstrated that liraglutide inhibits hepatic DNL (319), suggesting additional benefits beyond lowering body weight. Semaglutide, another GLP1 receptor agonist, has more pronounced body weight-lowering effects (320). In a phase II trial including patients with biopsy-confirmed NASH and fibrosis, semaglutide administered for 72 weeks led to a 13% reduction in body weight and was associated with higher rates of NASH resolution and improvement of fibrosis (321). With regards to ASCVD, liraglutide administered to patients with T2D has been consistently reported to improve circulating lipid profile (reduce triglycerides, total and LDL-cholesterol, and increase HDL-cholesterol) and reduce carotid IMT (322–324). The effects of semaglutide on atherosclerosis are currently being evaluated in phase IV trials (NCT03985384). Together, the above studies indicate the potential of GLP1 receptor agonists for concurrent treatment of NASH and ASCVD, which should be confirmed in long-term studies assessing cardiovascular outcomes in patients with NASH. Furthermore, considering that the expression of GLP1 receptor is not detected in livers (325, 326) and aortas (327) from mice, monkeys and humans, the mechanisms by which GLP1 receptor agonists protect against NASH and ASCVD, beyond lowering body weight, warrant further investigation.

SGLTs are membrane proteins that regulate nutrient transport across the intestinal epithelium and the proximal renal tubules. While SGLT1 is expressed primarily in enterocytes and absorbs glucose from the gut lumen, SGLT2 is expressed in the proximal tubule and regulates glucose reabsorption from the glomerular filtrate (328). Thus, by decreasing renal glucose reabsorption and increasing urinary glucose excretion, SGLT2 inhibitors, such as empagliflozin, reduce hyperglycemia in patients with T2D (329). Empagliflozin has been evaluated for NAFLD treatment in phase IV trials (NCT02637973, NCT02686476, NCT02964715). In patients with T2D, empagliflozin administrated for 20 weeks reduced circulating ALT and liver fat assessed by MRI-derived proton density fat fraction (MRI-PDFF) (330). Although including a small sample size (*n* = 9), a study in patients with T2D and biopsy-proven NASH reported that empagliflozin treatment for 24 weeks improved histological components of NASH including steatosis, ballooning and fibrosis while reducing blood glucose, body weight and total cholesterol (331). Dapagliflozin, another SGLT2 inhibitor given to patients with T2D and NAFLD for 12 weeks, lowered circulating ALT and AST together with glucose and body weight. However, compared with placebo, reduction in hepatic fat was found when dapagliflozin was combined with omega-3 carboxylic acids, but not as a monotherapy (332). Also, although lowering body weight, dapagliflozin administered to insulin-resistant overweight/obese individuals for 12 weeks did not improve hepatic steatosis (333). However, when given to patients with T2D and NAFLD for 24 weeks, dapagliflozin lowered circulating ALT, hepatic steatosis and fibrosis assessed by MRI-PDFF and magnetic resonance elastography (MRE) (334). Interestingly, a recent phase II study including patients with NASH reported that 12 weeks of treatment with licogliflozin, a dual SGLT1/2 inhibitor, reduced circulating ALT and hepatic fat assessed by MRI-PDFF (335). Importantly, dramatic beneficial cardiovascular outcomes have been reported in T2D patients treated with SGLT2 inhibitors. In long-term and large phase III trials including patients with T2D with or at risk for ASCVD, treatment with empagliflozin or dapagliflozin was associated with lower rates of cardiovascular death (336, 337). Considering that SGLT2 is primarily expressed in the kidneys, the mechanisms by which SGLT2 inhibitors reduce the cardiovascular risk and directly affect the atherosclerotic plaque, beyond glucose- and body weight-lowering effects, are not completely clear (338, 339). Furthermore, whether long-term treatment with SGLT2 inhibitors concurrently lowers NASH and ASCVD remains unknown.

### 5.2. Targeting lipid metabolism for simultaneous treatment of NASH and ASCVD: Challenges and opportunities

Lipid overload is central to the pathogenesis of NASH. Fatty acids are supplied in excess to the liver *via* 1) enhanced flow from lipolysis of triglycerides in adipose tissue, and 2) increased synthesis from carbohydrates, primarily fructose, *via* DNL (50, 340). In addition to increased lipogenesis, fructose also suppresses hepatic FAO (109). Enhanced DNL coupled with impaired FAO result in the formation of lipotoxic species that induce hepatic oxidative stress, proinflammatory and profibrotic responses to promote NASH (50, 341, 342). Therefore, pharmacological strategies aimed at inhibiting DNL or enhancing FAO can reduce hepatic lipotoxicity and attenuate NASH. Nevertheless, considering the major role of the liver as a regulator of systemic lipids, such approaches may have detrimental or beneficial effects on circulating lipids that may affect ASCVD.

In the initial step of fatty acid biosynthesis, acetyl-CoA is converted to malonyl-CoA by ACC (343). In phase II trials, patients with NASH treated for 12 weeks with the ACC inhibitor, firsocostat (GS-0976), showed reduced circulating ALT, hepatic steatosis and markers of fibrosis (344) mediated by inhibition of hepatic DNL assessed by heavy water labeling (345). However, similar to other ACC inhibitors [MK-4074 (346) or PF-05221304 (347)] treatment with firsocostat increased circulating triglycerides (344), which can be attributed to the upregulation of hepatic SREBP-1, enhanced VLDL production and impaired triglyceride clearance (348). While these findings raise concerns that targeting ACC may aggravate atherogenic dyslipidemia, co-administration of PF-05221304 with a diacylglycerol acyltransferase 2 inhibitor (PF-06865571), reduced liver fat assessed by MRI-PDFF and mitigated the increase in circulating triglycerides in patients with NAFLD (347). Nevertheless, the cardiovascular consequences of ACC inhibition either as a monotherapy or in combination with other drugs warrant further research in long-term clinical trials.

The conversion of acetyl-CoA and malonyl-CoA to palmitate is catalyzed by FAS, which controls the liver capacity to synthesize fatty acids through DNL (349). In a phase IIa trial including individuals with hepatic steatosis and fibrosis, treatment for 12 weeks with a FAS inhibitor, TVB-2640, dose-dependently decreased circulating ALT, AST and liver fat determined by MRI-PDFF. Importantly, TVB-2640 treatment significantly decreased circulating total and LDL-cholesterol. Although HDL-cholesterol was also decreased, lipidomics revealed beneficial effects including reduced triglycerides enriched in palmitate-containing species, diacylglycerols, bile acids and ceramides (350). Therefore, apart from the decrease in HDL-cholesterol, improved circulating lipid profile, reduced markers of hepatic steatosis and injury, indicate TVB-2640 as a promising candidate for dual treatment of NASH and ASCVD. Currently, TVB-2640 is evaluated in a phase IIb trial recruiting patients with NASH that will be treated for 52 weeks (NCT04906421). Longer-term studies are needed to determine the cardiovascular outcomes of TVB-2640 in patients with NASH.

The rate-limiting step in the synthesis of monounsaturated fatty acids is catalyzed by stearoyl-CoA desaturase 1 (SCD1) (351). The partial inhibitor of hepatic SCD1, aramchol, is a conjugate of cholic acid and arachidic acid, and is currently the most advanced drug candidate for NASH among those targeting hepatic DNL. In a 52-weeks, phase IIb trial including 247 patients with NASH, aramchol led to a time- and dose-dependent reduction in circulating ALT and AST. Histological analysis revealed that treatment with aramchol was associated with higher rates of NASH resolution and improvement in fibrosis compared with placebo (352). Of note, no significant differences in circulating lipid profile were found between the groups (352, 353). While the cardiovascular outcomes of SCD1 inhibition have not been addressed in humans, loss of SCD1 in *Ldlr*^−/−^ mice (354) or its inhibition in *Ldlr*^−/−^ / *Apob* 100/100 mice *via* ASO (355) enhanced atherosclerosis while reducing hepatic steatosis. Plans to test aramchol in the phase III/IV ARMOR trial (NCT04104321) in patients with biopsy-proven NASH and fibrosis for 5 years will shed light on the long-term effects of aramchol treatment on NASH and perhaps its cardiovascular consequences.

In addition to DNL inhibition, drugs that promote FAO can also lower hepatic lipotoxicity and NASH. This approach has been pursued by activation of key regulators of hepatic FAO, mainly PPARɑ and PPARβ/δ. Among the three PPAR isotypes (PPARα, PPARβ/δ and PPARγ), PPARα is the most abundant in hepatocytes where it acts as a master regulator of mitochondrial/peroxisomal FAO (356). In mice, hepatocyte-specific loss of PPARα enhances steatohepatitis, which is aggravated in whole-body *Ppara*^−/−^ mice, indicating a protective role for both hepatic and extrahepatic PPARα in NASH (357–359). Accordingly, the PPARα agonist, Wy-14,643, lowers MCD diet-induced NASH and fibrosis in mice (360). Few clinical studies evaluated the effects of the PPARα agonists, fibrates, in NASH. In patients with biopsy-confirmed NASH, treatment with fenofibrate for 48 weeks reduced circulating transaminases, triglycerides and glucose while increasing apolipoprotein A1. Histological assessment revealed improved hepatocellular ballooning, but no significant changes in steatosis, inflammation, and fibrosis (361). Interestingly, in patients with NASH and fibrosis, fenofibrate administered 2 weeks before the addition of the ACC inhibitor, firsocostat, not only mitigated hypertriglyceridemia, but also improved liver biochemistry compared to icosapent ethyl (Vascepa) (362). Pemafibrate, a selective PPARα modulator, lowers NASH in mice fed the MCD or AMLN diet (363). In a phase II trial including 117 patients with NAFLD, pemafibrate administered for 48 weeks lowered circulating ALT and LDL-cholesterol. Although liver fat assessed by MRI-PDFF was not altered, MRE-based liver stiffness was significantly reduced (364). The concurrent improvement in plasma lipids and liver biochemistry suggest beneficial effects of PPARα agonism in both NASH and ASCVD. Although this notion was supported by studies in *Apoe*^−/−^ (365) and ApoE*3Leiden mice (366) in which fenofibrate reduced atherosclerosis, a multinational trial including over 10,000 patients with CVD, demonstrated that pemafibrate was not associated with lower incidence of cardiovascular events although NAFLD incidence was reduced (153).

PPARβ/δ is ubiquitously expressed, including in hepatocytes, Kupffer cells and hepatic stellate cells (367, 368). Studies in mice lacking PPARβ/δ indicated its roles in regulating hepatic FAO and antiinflammatory responses in Kupffer cells (369, 370). The dual PPARα/δ agonist, elafibranor (GFT505), showed promising outcomes in preclinical NASH models (371) and in a phase IIb trial (372) in which 52 weeks of treatment with elafibranor led to higher rates of NASH resolution and reduction in fibrosis. Importantly, elafibranor not only decreased circulating transaminases, but also lowered triglycerides and LDL-cholesterol, increased HDL-cholesterol and improved glycemic control, indicating significant improvement of overall cardiometabolic risk (372). These promising findings led to the evaluation of elafibranor in a phase III trial (RESOLVE IT) including over 2,000 patients with histologically confirmed NASH (NCT02704403). Unfortunately, results of the week 72 interim analysis revealed that elafibranor did not achieve NASH resolution without worsening of fibrosis, and the RESOLVE-IT trial was discontinued.

The beneficial effects of elafibranor and the PPARγ agonist, pioglitazone, have raised interest in pan-PPAR agonism as a potential therapy for NASH. In preclinical studies, selective PPARα (fenofibrate), PPARγ (pioglitazone) and PPAR*δ* (GW501516) were compared to the pan-PPAR agonist, lanifibranor, and indicated that pan-PPAR agonism lowers experimental NASH by combining the beneficial effects of the three PPAR isotypes (373). Indeed, in a phase IIb trial including 247 patients with biopsy-proven NASH, lanifibranor administered for 24 weeks led to higher rates of NASH resolution and improvement in fibrosis compared with placebo. Importantly, in addition to lowering circulating transaminases, lanifibranor had beneficial effects on plasma lipid profile and glycemic control. Nevertheless, a mild increase in body weight (≈3%) was noted (374). Currently, the phase 3 NATiV3 trial (NCT04849728) is recruiting patients with NASH and fibrosis to assess the long-term efficacy of lanifibranor for up to 7 years. Findings from this study will provide important insight of the cardiometabolic consequences of pan-PPAR agonism in patients with NASH.

Statins reduce circulating cholesterol through inhibition of HMG-CoA reductase and remain the leading therapeutic in reducing the risk of cardiovascular events (375). Although dyslipidemia is a hallmark of both NAFLD/NASH and atherosclerosis, whether cholesterol-lowering by statin therapy improves NASH outcome remains inconsistent and thus is not a current recommendation for NASH management (376). Despite this, statin therapy may have pleotropic beneficial effects for the treatment of NAFLD/NASH. In MCD diet-fed mice, fluvastatin reduces hepatic steatosis and improves inflammation and fibrosis through activation of PPARɑ and its target genes enhancing FAO (377). Rosuvastatin blunts NASH-induced pro-inflammatory cytokine expression in livers from high-fat diet-fed STAM mice (378), while simvastatin reduces inflammation and fibrosis in *Apoe*^−/−^ mice fed a high-fat, high-cholesterol diet for 7 weeks with corresponding inhibition of Ras and Rho signaling (379). Treating obese mice with atorvastatin reduces cholesterol accumulation in isolated hepatocytes and reduces cholesterol-induced mitochondrial depletion of GSH (94), and atorvastatin is currently being evaluated in phase II trials for the treatment of NAFLD/NASH (NCT04679376). However, high-intensity atorvastatin therapy appears to enhance insulin secretion in patients with an increased risk of developing T2D (380). Since hyperinsulinemia is an early marker for metabolic disease (381) and is strongly associated with NAFLD (121), chronic use of statins in the treatment of NASH and ASCVD warrants further investigation with potential contraindications. Furthermore, statin users appear to have higher caloric intake, which is associated with weight gain and complicates disease progression (382).

A potential complicating factor is the presence of genetic variants or single-nucleotide polymorphisms (SNPs). In particular, SNPs in patatin-like phospholipase domain-containing protein 3 (*PNPLA3*), or transmembrane 6 super family 2 (*TM6SF2*) are known as strong predictors of NAFLD risk independent of associated metabolic confounding factors, despite these variants promoting lipotoxicity (383). However, the presence of the *PNPLA* and *TM6SF2* variants reduces the risk of ASCVD in patients with NAFLD (384). In contrast, mutations in Angiopoietin-like 3 (*ANGPTL3*) lead to hypolipidemia (385), since circulating ANGPTL3 inhibits lipoprotein lipase and is positively associated with NASH (386). Thus, therapeutics targeting PNPLA3, but not ANGPTL3, may be contraindicated should the outcome yield exacerbated ASCVD. These studies highlight the importance of identifying and considering genetic factors in both NAFLD and ASCVD which has been thoroughly discussed previously (14).

### 5.3. Ectopic fat as a potential link and therapeutic target in NAFLD/NASH and CVD

Patients with metabolic disease and obesity who have undergone bariatric surgery have marked improvement in insulin resistance (NCT03853590) and reduced risk of major cardiovascular events (387). Since previous studies demonstrated an association between bariatric surgery-induced weight loss and improved hepatic inflammation and fibrosis (388), a retrospective cohort study of nearly 1,200 patients with NAFLD and obesity was analyzed following bariatric surgery (389). Patients who received gastric bypass or sleeve gastrectomy demonstrated marked improvement in both adverse liver and cardiovascular outcomes (389). Since bariatric surgery effectively achieves weight loss in obese patients (390), the relationship between the effects of bariatric surgery and improved NASH and CVD outcome may be due to the effects of reducing visceral and ectopic adipose tissue. Although the risk of NASH and CVD rise with increasing BMI (391), ectopic fat [the storage of fat in non-adipose tissues (392)] and visceral fat [the storage of fat in the mediastinal and abdominal cavities (393)] appear to be a more reliable correlation between cardiometabolic disease compared with BMI alone (394). Similarly, CAD patients with normal BMI have enhanced visceral fat accumulation (395). Indeed, NAFLD patients with normal BMI have excessive visceral fat compared with non-NAFLD patients (396). The detrimental correlations between visceral fat, NASH and CVD are likely due in part to adipokine secretion, like TNFɑ (397), which mediates inflammatory responses locally and systemically. Independent of BMI, reducing visceral fat improves comorbidities of CVD and NASH (398). Consistent with this, calorie restriction improves NAFLD-related biomarkers such as transaminases, liver steatosis and fibrosis scores (399), as well as reducing the risk for atherosclerosis (400). The benefits for calorie restriction and improvement of NASH and ASCVD are multifactorial. Calorie restriction (1) reduces adipokine release which attenuates systemic inflammatory signaling (401), (2) reduces serum lipids and comorbidities associated with disease exacerbation (e.g., hypertension) (400), (3) activates autophagy, which protects against hepatic steatosis and inflammation (402), and (4) activates various molecular pathways (e.g., AMPK) which are associated with protection against NASH and atherosclerosis (403, 404).

AMPK responds to energy demand by sensing the ratio of ATP to ADP/AMP. Activation of AMPK enhances catabolism and reduces anabolism, but additionally protects against oxidative stress-induced endothelial activation in atherosclerosis (405). AMPK additionally augments reverse cholesterol transport in atherosclerosis and polarizes macrophages to an M2 phenotype (404), which are associated with plaque stability and regression (406). In murine models of NAFLD, AMPK activation is inhibited due to overnutrition (407). Thus, activation of AMPK yields improvement in both CVD and NASH outcome in mouse models. Metformin activates AMPK, which reduces hepatic steatosis (408), and activation of AMPK with PF-06409577 reduces dyslipidemia and liver transaminases in rats and non-human primates (409). Another AMPK activator, PXL770, attenuates DNL, hepatic steatosis, inflammation, and ballooning in mouse NASH models (403). These effects may be due to inhibition of mTORC1, which is inhibited by AMPK through phosphorylation of raptor (410). mTOR activates lipogenesis by inducing SREBP-1c activation (411). Selective inhibition of mTORC1 by folliculin (FCLN) deletion protects against NASH by TFE3 transcription factor-induced inhibition of lipogenesis (412); however, the impact of FCLN deletion has not been investigated in atherosclerotic mice. While clinical trials for NASH are ongoing and activation of AMPK by PXL770 in mice improves atherosclerotic outcome (403, 413, 414), whether these results extend to human atherosclerotic patients has yet to be explored.

### 5.4. Lowering inflammation for dual-targeting of NASH and ASCVD

Since primary components of the pathophysiology of NASH and atherosclerosis involve the regulation of inflammatory cytokines, leukocyte response, and the crosstalk between these mediators (300, 415), systemic therapy reducing inflammation may yield benefits across both pathologies. Given the potent effects of IL-1β signaling and its central role in inflammation, the monoclonal antibody targeting IL-1β (canakinumab) has been implemented in the CANTOS phase III clinical trials (NCT01327846) for the treatment of CVD (416). It is well-established that the proinflammatory cytokine, IL-1β, activates endothelial cells to express adhesion molecules, secrete chemokines, and vSMC proliferation to augment atherogenesis (417). Furthermore, IL-1β gene expression increases in livers of mice fed a high-fat, high-cholesterol (1.25%) diet for 18 weeks, and deletion of IL-1β reduces steatosis, inflammation, ballooning, and fibrosis in these mice (418). Thus far, the CANTOS trial has proved promising since inhibition of IL-1β reduces the total number of serious cardiovascular events in patients with prior MI history (416); however, it has not examined whether IL-1β inhibition by canakinumab improves characteristics of NASH. Since deletion of IL-1β reduces steatohepatitis, and fibrosis in mice fed a NASH-inducing diet (418), further investigation on the effects of canakinumab in human NASH are warranted. Such studies should also consider the potential risk of infection or sepsis considering that treatment with canakinumab was found to be associated with a higher incidence of fatal infection in the CANTOS trial (419).

While the effects of IL-1β inhibition remain unexplored in NASH clinical trials, several preclinical and clinical studies have analyzed the potential benefits of targeting TNFα for NASH. Antibody therapy against TNFα yielded promising results with diet-induced NASH in mouse and rat models showing improvement in circulating AST, ALT, steatohepatitis, and fibrosis (420–422). However, retrospective studies of patients receiving anti-TNFα for immune-related diseases reported no reduction in the incidence of new onset NAFLD, NASH, or cirrhosis (423). While antagonism of TNFα with monoclonal antibodies yielded effective results in patients with rheumatoid arthritis (424) and inflammatory bowel disease (425), clinical trials for anti-TNFα in patients with chronic heart failure (RECOVER and RENAISSANCE) were terminated prematurely due to no observable benefit (426). Furthermore, the anti-TNFα monoclonal antibody CNTO5048 (CNT) enhanced plasma triglycerides, VLDL, and atherosclerosis in *Ldlr^−/−^* mice (427), suggesting the use of anti-TNFα antibodies for atherosclerosis may be contraindicated.

While the effects of targeting cytokines in NASH and ASCVD warrant further investigation, chemokine signaling appears to be a promising direction in targeting inflammation. Since mice fed a choline-deficient diet have enhanced hepatic CCR2 (60) and clinical trials demonstrated efficacy and safety with antagonism of CCR2 and CCR5 in patients with HIV (428), the CENTAUR clinical trial (NCT02217475) proceeded with the CCR2/CCR5 dual antagonist cenicriviroc over the course of 2 years (429). At the completion of this phase IIb trial, patients with NASH and fibrosis who received cenicriviroc demonstrated marked improvement in fibrosis without worsening of NASH (429). Despite the completion of phase II clinical trial, phase III (AURORA, NCT03028740) was terminated early due to lack of efficacy. CCL2 signals through its receptor CCR2, which is required for monocyte emigration from the bone marrow during an inflammatory response (430), and deletion of CCR2 significantly reduces atherosclerosis (431). Inhibition of CCR2 with the MLN1202 monoclonal antibody reduced the levels circulating CRP, a marker of cardiovascular risk (432). Although the effects of cenicreviroc on CVD were studied in an early clinical trial (NCT01474954), the trial was terminated due to low enrollment. Taken together, the effects of CCR2/CCR5 inhibition may improve some aspects of ASCVD; however, since activation of both CCR2 and CCR5 receptors directly activate hepatic stellate cells which promote hepatic fibrosis (433), further investigation on the effects of cenicreviroc on vSMCs are indicated to determine whether treatment affects atherosclerotic plaque stability.

### 5.5. Targeting fibrosis in NASH and atherosclerosis: A potential contraindication

Despite the numerous studies investigating the mechanisms behind NASH or ASCVD, those that utilize genetic manipulation often do so with subsequent onset of disease. These studies, while informative, may not be appropriate for identifying therapeutic targets since intervention of NASH and ASCVD does not occur until they become symptomatic. More advanced disease, both for NASH and ASCVD, involve the accumulation of fibrous tissue in the liver and vessels, respectively. In NASH, advanced fibrosis is correlated with worse prognosis (434). In contrast, fibrous or fibrocellular atherosclerotic plaques confer stability against plaque rupture and catastrophic events (435). Therefore, systemic targeting of fibrosis to improve NASH may be contraindicated for maintaining stable fibroatheromas. Currently, several clinical trials for the treatment of NASH seek to improve clinical outcome which includes lowering fibrosis (374, 436, 437). However, it is unknown whether these will affect cardiovascular morbidity and mortality.

PPARs regulate lipid homeostasis through transcriptional control of FAO and DNL (438). Since the relationship between dysregulated lipid metabolism and NASH or ASCVD is well-established, therapeutics targeting of PPARs may yield successful results. Indeed, the PPARγ agonist pioglitazone and PPARα fenofibrate reduced atherosclerosis and hepatic steatosis in mice lacking both ApoE and Farnesoid x receptor (FXR) (439), which modeled NASH and atherosclerosis simultaneously. FXR, a nuclear receptor responsible for bile acid and cholesterol synthesis, suppresses hepatic lipogenesis and VLDL assembly by attenuating SREBP1-c (440). In addition, FXR activity promotes PPARα transcription through binding directly to the PPARα reporter (441). Currently the non-steroidal FXR agonist Cilofexor is undergoing Phase II clinical trials for NASH treatment and has shown promising results in the reduction of hepatic steatosis, inflammation, and fibrosis (436). While deletion of FXR from *Apoe^−/−^* mice enhanced atherosclerosis (442), it remains unknown if the anti-fibrotic effects of FXR in stellate cells is conserved in plaque associated vSMCs. Since Cilofexor primarily acts in the intestine (443), it may reduce the potential side effects of systemic FXR activation; however, further investigation on its effects on atherosclerotic plaques is warranted. FXR additionally induces FGF-19, which enhances cholesterol efflux and HDL assembly through modulation of hepatic ABCA1 and ApoA1 (444). Administration of the FGF-19 analog NGM282 reduces atherosclerosis in *Apoe^−/−^* mice, enhances plasma HDL-cholesterol in healthy subjects (444), and improved NASH and fibrosis in phase II clinical trials (445). However, NGM282 increases plasma LDL-cholesterol (437), suggesting a potentially exacerbating factor in atherosclerosis. Furthermore, NGM282 reduces atherosclerotic fibrosis in mice (444), implicating the potential for plaque rupture with sustained therapy. In addition to NGM282, the synthetic bile acid obetacholic acid agonizes FXR and has been implemented in phase III clinical trials for the treatment of NASH (NCT02548351). Since patients receiving obetacholic acid have enhanced circulating VLDL and LDL but reduced circulating HDL (21), its administration in the context of atherosclerosis may be contraindicated. Since these studies terminated at 72 weeks (21), the long-term effects of obetacholic acid on atherosclerosis remain unknown. Overall, the long-term effects of these NASH/fibrosis-targeting drugs must consider the potential effects on atherosclerotic plaque instability.

## 6. Concluding remarks and future directions

In this review, we highlighted our current gaps in knowledge with particular emphasis on modelling both diseases, common biomarkers and potential therapeutics, and the potential caveats we currently face by targeting specific aspects of each disease. In the past decade cardiovascular-related mortality rates are steadily increasing concomitant with a rapid rise in obesity and NAFLD/NASH incidences (1, 2), currently afflicting one-third of the population worldwide (6). Despite this prevalence, no FDA-approved drugs exist for the treatment of NASH. Since NASH serves as an independent risk factor for ASCVD, and individuals with NASH are at a greater risk of ASCVD-related mortality compared with liver-related mortality (7–12, 139–141), further understanding of the link between these two diseases is clearly indicated (8). Future studies establishing accepted models of NASH and atherosclerosis will provide a translational understanding of the relationship between NASH and ASCVD. By identifying new biomarkers shared between NASH and ASCVD, early detection and intervention will help to reverse the incline in NASH- and ASCVD-related mortality. Lastly, clinical trials seeking an effective therapeutic for NASH must heavily consider the potential influences on atherosclerotic plaque burden.

## Author contributions

AF designed the graphic in Figure 1 and Table 1. AY and OR designed Figure 2 with Biorender. All authors contributed to drafting and editing of the review. All authors contributed to the article and approved the submitted version.
